# Case Report: Long-Term Follow-Up of Visceral Leishmaniasis and HIV Coinfected Patients Without Relapse: Lymphoproliferative Response After Stimulation with Soluble *Leishmania* Antigen

**DOI:** 10.3390/microorganisms13030686

**Published:** 2025-03-19

**Authors:** Begoña Monge-Maillo, Daniel Roger-Zapata, Fernando Dronda, Eugenia Carrillo, Javier Moreno, María Dolores Corbacho-Loarte, Diego Gayoso Cantero, Oihane Martín, Sandra Chamorro-Tojeiro, Jose A. Perez-Molina, Francesca Norman, Marta González-Sanz, Rogelio López-Vélez

**Affiliations:** 1 National Reference Unit for Tropical Diseases, Department of Infectious Diseases, Ramón y Cajal University Hospital, WHO Collaborating Center for Clinical Management of Leishmaniasis, Ramón y Cajal Research Institute (IRYCIS), 28034 Madrid, Spain; mdcorbacho@hotmail.com (M.D.C.-L.); gayosocan@gmail.com (D.G.C.); sandracht@gmail.com (S.C.-T.); ffnorman@gmail.com (F.N.); martagonzalezsanz@hotmail.com (M.G.-S.); rlopezvelez.hrc@salud.madrid.org (R.L.-V.); 2 CIBER de Enfermedades Infecciosas (CIBERINFEC), Instituto de Salud Carlos III, 28029 Madrid, Spain; fdronda@hotmail.com (F.D.); ecarrillo@isciii.es (E.C.); javier.moreno@isciii.es (J.M.); oihane.martin.s@gmail.com (O.M.); 3 Hospital Universitario del Sureste, 28500 Madrid, Spain; drzmedicina@gmail.com; 4 Department of Infectious Diseases, Ramón y Cajal University Hospital, IRICYS, 28034 Madrid, Spain; 5 Unidad de Leishmaniasis y Enfermedad de Chagas, WHO Collaborating Center for Leishmaniasis, Centro Nacional de Microbiología, Instituto de Salud Carlos III, 28029 Madrid, Spain; 6 Microbiology Department, Ramón y Cajal University Hospital, IRYCIS, 28034 Madrid, Spain; 7 University of Alcalá, 28801 Alcalá de Henares, Spain

**Keywords:** visceral leishmaniasis, HIV, lymphoproliferative response, relapse, secondary prevention, recurrence

## Abstract

Highly active antiretroviral therapy (HAART) has reduced the incidence of VL/HIV dramatically. However, HAART only partially prevents relapses, with one-year relapse rates ranging from 30 to 60%. Consequently, secondary prophylaxis is recommended for patients with <200 CD4+ cells/μL. In clinical practice, characterizing cellular immune response could help estimate the risk of relapse in VL/HIV coinfected patients. In this study, the lymphoproliferative response after stimulation with soluble *Leishmania* antigen was assessed in 2022 and 2023 in three cases of VL/HIV coinfection with long-term follow-up (17, 8 and 19 years). PCR and rK-39 results for *Leishmania*, HIV viral load, CD4 cell count, proliferation index, IFN-γ, IL-2, IP-10, IL-10 and TNF-α were determined. Heterogeneous results were obtained, with only one patient having developed specific cellular immunity against *Leishmania*. No cases of relapse were observed. The heterogeneity of lymphoproliferative test results in the three cases described highlights the need to identify surrogate markers of cure to guide maintenance or withdrawal of prophylaxis.

## 1. Introduction

Leishmaniasis is a group of diseases caused by the flagellate, obligate intracellular protozoan parasite *Leishmania*, transmitted by the bite of female sandflies of the *Phlebotomus* genus [1]. It belongs to the so-called group of neglected tropical diseases (NTDs). Whereas other forms of *Leishmania* are self-limiting or even asymptomatic, visceral leishmaniasis (VL) can be life-threatening. Indeed, if left untreated, VL is almost always fatal, particularly in HIV-coinfected patients [1].

It is estimated that 30,000 new cases of VL and over 1 million new cases of cutaneous Leishmaniasis (CL) occur annually worldwide [2,3]. In 2022, it was estimated that 39 million (33.1–45.7 million) people were living with HIV. Moreover, 1.3 million (1.0–1.7 million) new cases were diagnosed that year [4].

As of 2022, cases of *Leishmania*/HIV coinfection have been reported in 42 countries. During the 2014–2022 period, 3431 cases (new, relapses and unspecified) of VL/HIV coinfection were reported [2]. A recent systematic review and meta-analysis revealed a prevalence of 6% of visceral *Leishmania*/HIV coinfection in people living in or coming from leishmaniasis-endemic regions. The studies available report a higher prevalence in Asia and the Americas, as compared to Europe, although data were unavailable for other leishmaniasis-endemic regions such as Africa and the Middle East [5].

Specific *Leishmania*/HIV interactions at the cellular level may influence the course of the two infections. VL appears to impair immunological competence in HIV-positive patients and increase the HIV load. Concomitant VL and HIV infection is characterized by significantly lower rates of cure, higher rates of drug toxicity and higher relapse and mortality rates due to VL, as compared to HIV-negative VL patients [6].

The incidence of VL/HIV has decreased dramatically with the advent of highly active antiretroviral therapy (HAART). However, its effect on relapse is only partial, with one-year relapse rates ranging from 30 to 60%. Therefore, secondary prophylaxis is recommended for patients with <200 CD4+ cells/μL. Prophylactic therapy can be discontinued when CD4+ count has remained constant at 200 cells/μL for more than six months. However according to the evidence available, CD4 levels may not be an optimal predictor of VL recurrence [7,8], thereby making it difficult to establish the appropriate duration of prophylaxis.

In immunocompetent patients with VL, successful response to treatment relies on the activation of a Th1 subset of CD4+ *Leishmania*-specific T-cells and the production of IFN-Y [9]. Based on this, peripheral blood mononuclear cell (PBMC) proliferation after challenge with soluble *Leishmania* antigen (SLA) (the soluble *Leishmania* antigen cell proliferation assay [SLA-CPA]) [10,11] and increased IFN-Y secretion by these cells [12,13] have been highlighted as potential in vitro markers of treatment response [14]. In SLA-CPA, PBMC are cultured in vitro and exposed to SLA. Later, lymphoproliferative response is assessed by estimating the “proliferation index” and the levels of cytokines and chemokines in the supernatants. However, this assay is complex and requires the use of technical equipment. In contrast, the whole blood assay (WBA) is simpler. In WBA, aliquots of whole blood are incubated with SLA; subsequently, levels of cytokines and chemokines are measured in the supernatants (SLA-WBA) [14].

In this study, we assessed the cellular immune response against VL in three patients coinfected with HIV who underwent long-term follow-up involving secondary prophylaxis, and who experienced no relapse. Immune response in recently diagnosed and treated HIV-VL coinfected patients has been reported [15]. This is the first study to examine immune response years after diagnosis of VL/HIV coinfection.

## 2. Case Descriptions

The three patients included in the study were VL/HIV coinfected patients that underwent long-term follow-up at the Department of Infectious Diseases of the Ramon y Cajal Hospital in Madrid. Diagnosis and treatment of HIV and VL were indicated by the Department. Regular follow-up with anamnesis, physical examination and analytical control was performed.

At two time points, January 2022 and October 2023, specific cellular response to *Leishmania infantum* and microbiological *Leishmania* analysis was assessed by performing the following:

− Soluble *Leishmania* antigen cell proliferation assay (SLA-CPA). The results are shown as a proliferative stimulation index (PI).
− Soluble *Leishmania* antigen whole blood assay (SLA-WBA). Based on previous studies cytokine and chemokine were determined as follows: IFN-γ, TNF-α, IP-10, IL-2 and IL-10 [10,14].
− *Leishmania* DNA was detected by nested PCR.
− Antibody detection against *Leishmania* was performed using the dipstick format Kalazar Detect Rapid Test rK39 Immunochromatographic Test.

Regarding the immunological and microbiological parameters, the risk of relapse was estimated as follows:

− Negative parasitological and positive immunological biomarkers: LOW risk of relapse: FOLLOW-UP of VL symptoms and *Leishmania* PCR.
− Positive parasitological and negative immunological biomarkers: HIGH risk of relapse: SECONDARY PROPHYLAXIS.
− Parasitology negative and immunology negative biomarkers: MEDIUM risk of relapse: FOLLOW-UP. VL symptoms and *Leishmania* PCR.

### 2.1. Case 1

**Figure 1:**
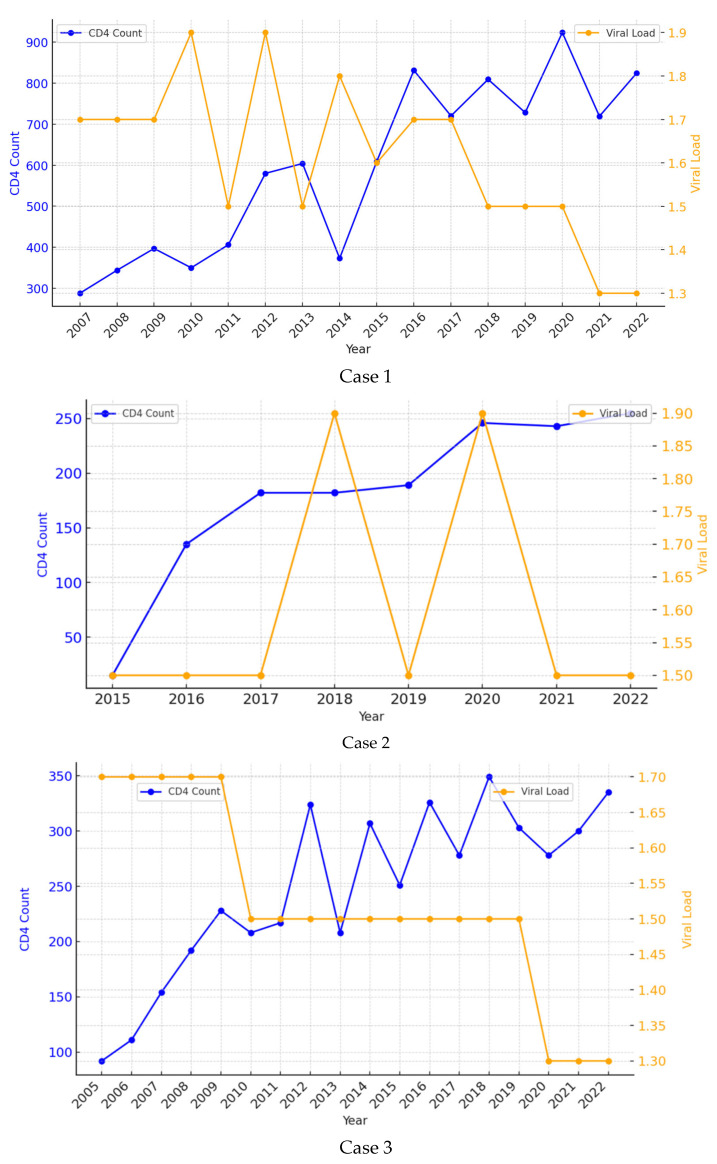
Evolution of CD4 cell account and HIV viral load during the follow up period of the three patients.

A 38-year-old female patient was referred to our Inpatient Department in June 2006 with complaints of marked asthenia, fever, weight loss and diarrhea over the last few weeks. She had no other medical history of interest. During the first days of admission, she was diagnosed with VL (visualization of amastigotes in bone marrow aspirate) and HIV coinfection with a viral load of 141,250 copies/mm^3^ and a CD4 count of 122 cells/mL. She was initially treated with pentavalent antimonials, which were soon replaced with liposomal amphotericin B (LAB) due to pancreatic toxicity, at a total dose of 1500 mg (35 mg/kg regimen). At the end of treatment, she met the criteria for a complete clinical cure. She received three doses of secondary prophylaxis with LAB every two weeks for two months. HAART was started a few weeks after completion of the VL treatment with good adherence and a favorable virological and immune response. The patient has remained with an undetectable viral load and a CD4 count persistently above 200 cells/mL since 2008. She has never presented with symptoms suggestive of VL recurrence during 17 years of follow-up (Figure 1—Patient 1).

Table 1 summarizes the results of the first lymphoproliferative study performed in January 2022. The patient had the highest CD4 count (734 cell/mL), an undetectable HIV viral load, and PCR and rK39 tests were negative for *Leishmania*. However, levels of PI, IFN-γ, IL-2, IP-10, IL-10 and TNF-α were below the cut-off value. We concluded that her specific cellular immunological response to *Leishmania* was negative. She was determined to have an intermediate risk of relapse.

Table 2 summarizes the results of the second lymphoproliferative study performed in October 2023. The patient continued being the one with the highest CD4 count (1009 cells/mL) and PI (1799), and an undetectable HIV viral load. PCR and rK39 tests were negative for *Leishmania*, with no specific cellular immunological response. She remains at an intermediate risk of relapse.

### 2.2. Case 2

A 54-year-old man presented to our Emergency Department with fever, pancytopenia and splenomegaly in March 2015. He had been diagnosed with HIV 28 years before with poor adherence to treatment. VL coinfection was confirmed by visualization of the parasite in the bone marrow aspirate and a positive peripheral blood PCR. At that time, his CD4 count was 8 cells/mL, with a viral load of 234,420 c/mm^3^. He completed treatment with LAB (total dose 3000 mg, in a 40 mg/kg regimen), after which he met the criteria for a complete clinical cure. HAART was started on admission, with good adherence. During follow-up, he developed a complete virological response, but his immune response was only partial, with CD4 levels consistently around 150 cells/mL over the years. Secondary prophylaxis with LAB 300 mg was administered monthly for five years (2015–2020) until DC4 was >200 cells/mL with good tolerance. To date and after eight years of follow-up, there is no clinical evidence of relapse (Figure 1—Case 2).

Table 1 summarizes the results of the first lymphoproliferative study, performed in January 2022. His CD4 count was significantly lower, as compared to the other two patients (<200 cell/mL) with an undetectable HIV viral load, and negative PCR and rK39 results for *Leishmania*. He presented PI below the cut-off value, but IFN-γ, IL-2, IP-10, IL-10 and TNF-α were detected. He therefore remains at an intermediate–low risk of relapse.

Table 2 summarizes the results of the second lymphoproliferative study performed in October 2023. The patient presented an increase in CD4 cell/mL, whereas the HIV viral load remained undetectable and PCR and rK39 results continued being negative for *Leishmania*. This time, the patient presented a positive PI and IFN-γ. IP-10, and TNF-α were also detected. He therefore remains at a low risk of relapse.

### 2.3. Case 3

A 50-year-old man was admitted to our Inpatient Unit with fever and pancytopenia in October 2004. He was diagnosed with HIV infection and VL by visualization of amastigotes in a bone marrow biopsy. Regarding HIV, he had a CD4 count of 70 cells/mL and a viral load of 251,188 copies/mL. Treatment with LAB was started at a total dose of 2000 mg (40 mg/kg regimen). At the end of treatment, the patient met the clinical and parasitological criteria for cure. He maintained good adherence to HAART and achieved an undetectable viral load and a CD4+ count of around 330 cells/mL. He received LAB prophylaxis at 300 mg monthly for 7 years (2005 to 2012), which was discontinued when DC4 was >200 cells/mL. At no time over 19 years of follow-up has he presented with findings compatible with VL recurrence (Figure 1—Patient 3).

Table 1 summarizes the results of the first lymphoproliferative test, performed in January 2022. He had a CD4 cell count above 200 cells/mL, an undetectable HIV viral load and negative PCR and rK39 results for *Leishmania*. Despite his low response to PI, IL-2 was significantly elevated, and IL-10 levels were also above the cut-off value. He therefore remains at an intermediate–low risk of relapse.

Table 2 summarizes the results of the second lymphoproliferative study, performed in October 2023. His CD4 cell count had decreased but remained above 200 cells/mL, with an undetectable HIV viral load. PI was still negative, IL-2 was elevated but IL-10 was now negative. He therefore remains at an intermediate–low risk of relapse.

## 3. Discussion

This study describes the course of three cases of VL and HIV coinfection over long-term follow-up (17, 8 and 19 years) with liposomal amphotericin B, both as the therapy of choice and as secondary prophylaxis. They showed good tolerance to regular administration of LAB even after seven years of prophylaxis, as described in Case 3. None of the patients experienced relapse during follow-up.

*Leishmania* parasites and HIV have reciprocal modulation in their pathogenesis. They both interact with specific antigen-presenting cells (macrophages and dendritic cells) by disrupting their normal function and preventing intracellular pathogen killing [16]. *Leishmania* can also modulate the HIV life cycle in various ways which can potentially lead to poor HIV load control, thereby resulting in an impaired CD4 cell count response [17,18,19]. In fact, in Cases 2 and 3, almost five years had passed before the CD4 cell count exceeded 200 cells/mL.

There is cumulative evidence that the host immune response is critical in VL treatment response and control, acting in synergy with anti-*Leishmania* drugs [20,21]. This implies that in immunosuppressed individuals, targeting parasites alone with conventional anti-*Leishmania* drugs without enhancing the immune response might simply not be sufficient [22] and therefore increase the risk of relapse [23]. In HIV and VL coinfected patients, many factors have been associated with VL relapse but in clinical practice, CD4+ cell count is the most widely used marker of the risk of recurrence. Hence, a count of <200 cells/μL has been established as the cut-off value for the administration of secondary prophylaxis [19]. Other factors such as HAART therapy compliance and viral load do not seem to be good predictors [18,24].

In our study, Cases 2 and 3 survived for at least five years with a CD4 cell count < 200 cells/mL. A systematic review revealed average rates of relapse for patients not receiving secondary prophylaxis of 67% vs. 31% for those receiving prophylaxis [25]. Therefore, the correct and systematic administration of secondary prophylaxis was most probably involved in the absence of relapse after so many years of follow-up.

The resolution of visceral *Leishmania* depends on adequate T-cell activation, particularly a strong Th1 response to eliminate intracellular pathogens. This is associated with the production of IL-12, IL-8 and especially IFN-γ [26]. There is strong evidence supporting that the lack of production of IFN-γ is an important risk factor for the development of VL. HIV infection modifies T-cell response to *Leishmania*, resulting in an overproduction of Th2-associated cytokines, including IL-4, IL-5 and, remarkably, IL-10. This molecule downregulates the production of IFN-γ and IL-12 and reduces the *Leishmania* killing activity of macrophages [27]

Lymphoproliferative response after SLA, as an immunological marker of good *Leishmania*-specific Th1 response after infection, could be a predictor of cure and indicate no risk of relapse after a first episode of visceral leishmaniasis [28]. Moreover, in previous studies, IP-10, and IFN-γ levels proved useful in monitoring the cellular immune response following treatment for active disease [14] and IFN-γ, TNF-α, IL-10 and IL-2 indicated exposure to leishmaniasis [10]. In clinical practice, lymphoproliferative response and cytokines could provide guidance about when to initiate or maintain secondary prophylaxis.

To the best of our knowledge, the only evidence available for *L. infantum* to date was provided by a study in ten patients with VL and HIV in Madrid, Spain. Six of the ten patients had a positive SLA cell proliferation test. Three patients with a positive SLA cell proliferation test were not receiving prophylaxis. Secondary prophylaxis was discontinued in the other three patients on the basis of their test results (CD4 counts: 152, 189, 359). No cases of relapse were observed during a mean follow-up period of 60 weeks. It is interesting to note that when prophylaxis was discontinued, two of the six patients with an adequate cellular response had less than 200 CD4 cells/μL, and no relapse was reported [15]. The current study provides new evidence of the applicability of SCA for monitoring the course of HIV and VL coinfection, with the particularity of the long follow-up period, added to the assessment of cellular immunity against *Leishmania* in HIV patients after years free of relapse.

In the first lymphoproliferative study carried out in 2022, none of the patients had a positive PI. However, Cases 2 and 3 expressed cytokines: IFN-γ, IL-2, IP-10, IL-10, TNF-α in Case 2 and IL-2 and IL-10 in Case 3. These results suggest a partial cellular immune response. The PI cut-off value used for cured immunocompetent patients (2.0) has been suggested to be lowered for coinfected patients with some degree of immunosuppression.

In the second lymphoproliferative study, it is noticeable that Case 2 developed a stronger cellular immunity against *Leishmania* with a positive PI. In contrast, cytokine expression decreased in Case 3, whereas no changes were observed in Case 1. These changes show that cellular immune response to *Leishmania* is a dynamic process in constant evolution influenced by many factors. This has been reported in other types of immunocompromised patients [29].

The risk of relapse in the three patients as assessed based on the results of the lymphoproliferation study raises some questions. Firstly, despite the weak lymphoproliferative response of all patients, especially in Case 1, none experienced relapse, and their PCR results were consistently negative for *Leishmania*. Case 1 was considered to have an intermediate risk of relapse as she may eventually develop a cellular response or lose her weak immunity and ultimately develop the disease.

On the other hand, the fact that Cases 2 and 3 did not show PI but expressed cytokines shows that PI may not be detected in some immunosuppressed patients. The reason is that the few cells that proliferate may still be capable of producing a significant amount of cytokines that can be detected in the supernatant. This way, it is possible that in immunosuppressed patients, the presence of certain clones of *Leishmania*-specific T-cells, even if they are few, is sufficient to protect the patient. In Cases 2 and 3, we could consider that their risk of relapse is low. It also is worth noting that the patient who maintained a significantly higher CD4 count from the outset is (Case 1) the one who did not develop cellular immunity against *Leishmania*.

Based on previous results [15], specific immunity against *Leishmania* is probably not solely related to the CD4 count but also to CD4 functional activity, as assessed by the SLA cell proliferation test. Hence, in these three cases probably a small number of memory T-cells could be enough for the patient to develop an effective secondary immune response. In addition, other factors are likely to be involved in the absence of relapse in these three patients, such as the type of HAART and good adherence to it throughout the follow-up period [30,31]. The pathogenesis and virulence of the Leishmania strain involved may also play a role.

A limitation of this study is the small number of patients and the need for a longer period of follow-up with new cellular response studies to determine the possible heterogeneity of its evolution. A protocol is needed for the correct timing and periodicity of SCA cell proliferation testing in HIV/VL infected patients. In addition, the validity of the cut-off values for PI and cytokines should be reconsidered in immunocompromised patients. And finally, to determine the risk of relapse and need for prophylaxis, it is necessary to establish the levels and combinations of cytokines that indicate an effective immune cell proliferative response to SLA when PI is undetectable. These values should certainly be weighed together with *Leishmania* PCR negativity, CD4 levels and HIV viral load. Moreover, further studies assessing CA cell proliferation during the follow-up of patients with HIV and VL coinfection may help develop new therapeutic approaches that target host immunity. This would help prioritize the target molecule for the development of immunomodulatory interventions for patients unresponsive to conventional therapies persistently developing relapse despite secondary prophylaxis [32].

## 4. Conclusions

The three cases described in this study highlight the heterogeneity of lymphoproliferative test results after stimulation with soluble *Leishmania* antigen in patients with long-standing VL/HIV coinfection. Although the proliferative stimulation test was positive in only one case, the three remained free of symptomatic VL during the follow-up period. This suggests that other biomarkers and cytokines may play an important role in maintaining the necessary immunity to avoid relapse. The immunological status induced by coinfection should be further investigated. Intervention studies are needed to identify surrogate markers of cure and protection.

## Figures and Tables

**Table 1:** Results of the HIV control data, microbiological analysis of *Leishmania* and proliferative index and cytokines levels after stimulation with soluble *Leishmania* antigen SLA in three patients (January 2022).

	HIV RNA	CD4	PCR Leish	rK39	CPA	IFN-Y	IL-2	IP-10	IL-10	TNF-α
	(Copies/mL)	(n/mL)			PI	(pg/mL)	(pg/mL)	(pg/mL)	(pg/mL)	(pg/mL)
Patient 1	<20	732	NEG	NEG	1.799	0	0	139.02	0	8.47
					NEG	NEG	NEG	NEG	NEG	NEG
Patient 2	<20	150	NEG	NEG	1.652	100.15	2500	1293.56	51.18	113.7
					NEG	POS	POS	POS	POS	POS
Patient 3	<20	330	NEG	NEG	1.160	30.70	760.69	440.27	17.12	11.50
					NEG	NEG	POS	NEG	POS	NEG

Positivity cut-off value for CPA and each cytokine/chemokine were established as follows: PI 2.0; IFN-Y 40.3 pg/mL; IL-2 19.1 pg/mL; IL-10 15 pg/mL; IP-10 1142 pg/mL; TNF-α 22.77 pg/mL.

**Table 2:** Results of the HIV control data, microbiological analysis of *Leishmania* and proliferative index and cytokines levels after stimulation with soluble *Leishmania* antigen SLA in three patients (October 2023).

	HIV RNA	CD4	PCR Leish	rK39	CPA	IFN-Y	IL-2	IP-10	IL-10	TNF-α
	(Copies/mL)	(n/mL)			PI	(pg/mL)	(pg/mL)	(pg/mL)	(pg/mL)	(pg/mL)
Patient 1	<20	1009	NEG	NEG	1.169	0	0	0	0	0.71
					NEG	NEG	NEG	NEG	NEG	NEG
Patient 2	<20	382	NEG	NEG	12.625	1754.56	ND	4243.52	1.86	1969.65
					POS	POS		POS	NEG	POS
Patient 3	<20	277	NEG	NEG	0.924	1.65	46.33	441.37	0	0
					NEG	NEG	POS	NEG	NEG	NEG

Positivity cut-off value for CPA and each cytokine/chemokine were established as follows: PI 2.0; IFN-Y 40.3 pg/mL; IL-2 19.1 pg/mL; IL-10 15 pg/mL; IP-10 1142 pg/mL; TNF-α 22.77 pg/mL. ND: Not done.

## Data Availability

The original contributions presented in this study are included in the article. Further inquiries can be directed to the corresponding author.

## Abbreviations

The following abbreviations are used in this manuscript:

VL	visceral leishmaniasis
HIV	human immunodeficiency virus
HAART	highly active antiretroviral therapy
CL	cutaneous Leishmaniasis
SLA	soluble Leishmania antigen
SLA-CPA	antigen cell proliferation assay
WBA	whole blood assay
